# Association of Lean Body Mass and Fat Mass With 1-Year Mortality Among Patients With Heart Failure

**DOI:** 10.3389/fcvm.2022.824628

**Published:** 2022-02-28

**Authors:** Yilan Ge, Jiamin Liu, Lihua Zhang, Yan Gao, Bin Wang, Xiuling Wang, Jing Li, Xin Zheng

**Affiliations:** ^1^National Center for Cardiovascular Diseases, National Clinical Research Center for Cardiovascular Diseases, NHC Key Laboratory of Clinical Research for Cardiovascular Medications, State Key Laboratory of Cardiovascular Disease, Fuwai Hospital, Chinese Academy of Medical Sciences and Peking Union Medical College, Beijing, China; ^2^National Clinical Research Center for Cardiovascular Diseases, Coronary Artery Disease Center, Fuwai Hospital, Chinese Academy of Medical Sciences, Shenzhen, China

**Keywords:** lean body mass, fat mass, body mass index, heart failure, mortality

## Abstract

**Background:**

Prior studies have found an unexplained inverse or U-shaped relationship between body mass index (BMI) and mortality in heart failure (HF) patients. However, little is known about the independent effects of each body component, i.e., lean body mass (LBM) and fat mass (FM), on mortality.

**Methods:**

We used data from the China Patient-centered Evaluative Assessment of Cardiac Events-Prospective Heart Failure Study. LBM and FM were calculated using equations developed from the National Health and Nutrition Examination Survey. LBM and FM index, calculated by dividing LBM or FM in kilograms by the square of height in meters, were used for analysis. We used restricted cubic spline and Cox model to examine the association of LBM and FM index with 1-year all-cause mortality.

**Results:**

Among 4,305 patients, median (interquartile range) age was 67 (57–76) years, 37.7% were women. During the 1-year follow-up, 691 (16.1%) patients died. After adjustments, LBM index was inversely associated with mortality in a linear way (*P*-overall association < 0.01; *P*-non-linearity = 0.52), but no association between FM index and mortality was observed (*P*-overall association = 0.19). Compared with patients in the 1st quartile of the LBM index, those in the 2nd, 3rd, and 4th quartiles had lower risk of death, with hazard ratio of 0.80 (95% CI 0.66–0.97), 0.65 (95% CI 0.52–0.83), and 0.61 (95% CI 0.45–0.82), respectively. In contrast, this association was not observed between FM index quartiles and mortality.

**Conclusion:**

Higher LBM, not FM, was associated with lower 1-year mortality among HF patients.

## Introduction

Obesity, as indexed by high body mass index (BMI), is a major risk factor for incident heart failure (HF) (1), and up to 60–80% of patients with HF are overweight or obese (2–4). However, in established HF, an unexpected inverse or *U*-shaped relation between BMI and mortality was consistently observed (2, 4–9). This paradoxical observation has been referred to as the “obesity paradox.”

One of the possible reasons underlying this obesity paradox is the inaccuracy of BMI in estimating body fat, as BMI is an aggregate of lean body mass (LBM) and fat mass (FM), and LBM and FM may act differently on mortality (10–15). Therefore, understanding different contributions of body compositions may facilitate understanding obesity paradox and providing valuable information for obesity management to improve the prognosis of HF patients. Direct measurement of body composition is high-cost and requires sophisticated technologies, which limits its utilization in clinical practice. Therefore, to date, few studies, with relatively small sample size, have reported the effect of body composition among HF patients (16–19). There is a need to evaluate this association in a large population to assure adequate statistic power. A much more practical method of measuring body composition, which can be conducted in large-scale study, is the prediction equations derived from anthropometric measurement, and this method has been validated against dual-energy x-ray absorptiometry (14, 20). However, to our knowledge, the association between predicted LBM and FM by anthropometric equations with HF prognosis is scarcely explored. In addition, whether the age, sex, and comorbidities may modify the effect of body composition on mortality remain largely unknown, subgroup analysis is warranted to reveal more information.

Accordingly, using previously validated anthropometric equations to estimate LBM and FM, we evaluated the independent roles of LBM and FM in relation to all-cause mortality among patients hospitalized for HF in a large prospective cohort study.

## Methods

### Study Design and Participants

The patients of this study were from a prospective cohort of acute HF, the China Patient-centered Evaluative Assessment of Cardiac Events-Prospective Heart Failure Study (China PEACE 5p-HF Study), which enrolled patients from 52 hospitals between 2016 and 2018 (21). We included patients aged 18 years or older who were hospitalized with a primary diagnosis of new-onset HF or decompensation of chronic HF; and excluded those who died during the index hospitalization (*n* = 32), or were lost to follow-up at 1 year after discharge (*n* = 7), or lacked information on height, waist circumference, or weight during hospitalization—these information was required to calculate predicted LBM and FM (*n* = 563), leaving 4,305 patients for analyses. All enrolled patients signed written informed consent. The China PEACE 5p-HF Study was approved by the Ethics Committees of Fuwai Hospital and all collaborating hospitals. The study was registered at www.clinicaltrials.gov (NCT02878811).

### Anthropometric Measures

BMI was analyzed according to weight and height at discharge using the formula weight (kg)/(height in m)^2^. Patients were stratified into the following categories recommended by the Working Group on Obesity in China according to BMI value: underweight (< 18.5 kg/m^2^), normal weight (18.5–23.9 kg/m^2^), overweight (24–27.9 kg/m^2^), and obese (≥ 28 kg/m^2^) (22).

LBM and FM were calculated using anthropometric prediction equations developed from the National Health and Nutrition Examination Survey (NHANES) (23), and the equations are shown in Supplementary Table 1. The high predictive ability for body compositions of these equations has been validated by several studies (14, 20). We calculated the LBM index (kg/m^2^) and FM index (kg/m^2^) using LBM and FM, respectively, in kilograms divided by the square of height in meters.

### Data Collection

Detailed information on demographics, clinical characteristics, comorbidities, and discharge medications were obtained through abstraction of medical charts and in-person interviews during the index hospitalization. Left ventricular ejection fraction (LVEF) was measured during the index hospitalization by trained physicians with a standard protocol. Blood samples of enrolled patients were taken within 48 h of admission to perform analysis in the central laboratory.

### Outcomes

The outcome of the study was all-cause death within 1 year after discharge. Information regarding patient survival status during 12 months of follow-up was collected from follow-up interviews, medical documents, and the national death cause database. All deaths were centrally adjudicated by trained clinicians.

### Statistical Analysis

Continuous variables were expressed as mean ± SD or medians with interquartile range (IQR) and compared by the Kruskal-Wallis test. Categorical variables were presented as frequencies with percentages and compared by the χ^2^-test.

The LBM index and FM index were categorized into sex-specific quartiles or were treated as continuous variables with the effect estimates for 1-SD increase. Cox proportional hazards models were used to estimate the hazard ratios (HRs) of the 1-year all-cause mortality associated with the LBM index and FM index. Separate Cox regression models were fitted with (1) model 1, no adjustment; (2) model 2, adjustment for age, sex, education level, systolic blood pressure at admission, heart rate at admission, NYHA class, LVEF, serum sodium, serum albumin, Hs-cTnT, NT-pro BNP, estimated glomerular filtration rate (eGFR), current smoking status, history of coronary heart disease, hypertension, chronic obstructive pulmonary disease, anemia, valvular heart disease, diabetes mellitus, atrial fibrillation and prescription of angiotensin-converting enzyme inhibitors (ACEIs)/ angiotensin receptor blockers (ARBs), β-blockers, and mineralocorticoid receptor antagonists (MRAs); (3) model 3, the LBM or FM index was added to model 2 for mutual adjustment. In order to test linearity assumption between body composition indices and mortality, restricted cubic splines (RCS) were used in multivariable-adjusted models.

We conducted interaction and stratified analyses to evaluate the potential effect modification. We also performed several sensitivity analyses. First, we used different categories for the LBM and FM index (thirds or fifths). Second, we tested the robustness of our findings by using another body composition prediction equation developed from Chinese population (24). Third, due to the possible influence of reverse causation, we performed sensitivity analyses by excluding patients who might be in severe medical condition, including patients who died during the first 3 months of follow-up, or who had a BMI < 18.5 kg/m^2^.

All statistical analysis was performed by SAS version 9.4 (SAS Institute, Cary, NC, USA) and R programming language version 3.6.0 (R foundation for Statistical Computing, Vienna, Austria). All comparisons were two-sided, and statistical significance was defined as *P* < 0.05.

## Results

### Population Characteristics

A total of 4,305 patients were included in the analysis. The median age of the study population was 67 (57–75) years, 37.3% were women, and 60.1% were overweight or obese patients. The median LBM indexes were 17.0 (15.5–18.5) and 13.9 (12.8–14.9) kg/m^2^, and the median FM indexes were 6.6 (5.1–8.1) and 9.4 (7.6–11.3) kg/m^2^ for men and women, respectively. Table 1 shows the baseline characteristics of participants according to sex-adjusted LBM index quartiles. Patients with low LBM index were more likely to be older, have a lower level of education, and have a history of valvular heart disease, chronic obstructive pulmonary disease, and anemia; were less likely to have a history of metabolic syndrome, hypertension and diabetes mellitus; had lower levels of systolic blood pressure, LDL, TG, waist circumference, BMI, FM index, plasma sodium, and albumin; had higher level of HDL; had more severe HF (i.e., a higher proportion of NYHA functional classes III to IV and increased hs-cTnT and NT-proBNP levels); and were less likely to receive ACEIs/ARBs while more likely to receive MRAs.

**Table 1:** Baseline characteristics of patients hospitalized for HF among sex-adjusted lean body mass (LBM) index quartiles.

	**Total**	**LBM index**				
		**The 1st quartile**	**The 2nd quartile**	**The 3rd quartile**	**The 4th quartile**	***P*-value**
***N***	**(*n* = 4,305)**	**(*n =* 1,075)**	**(*n* = 1,078)**	**(*n* = 1,076)**	**(*n* = 1,076)**	
Age, yr (IQR)	67 (57–75)	70 (62–78)	68 (60–76)	66 (56–74)	63 (51–72)	<0.01
Female, *n* (%)	1,607 (37.3)	401 (37.3)	403 (37.4)	401 (37.3)	402 (37.4)	1
High school education or above, *n* (%)	1,221 (28.4)	234 (21.8)	307 (28.5)	312 (29.0)	368 (34.2)	<0.01
**Clinical features**						
LVEF, % (IQR)	44.0 (33.0–57.0)	43.0 (32.0–56.0)	44.0 (33.0–57.0)	43.0 (33.0–56.0)	44.0 (34.0–58.0)	0.19
NYHA, *n* (%)						<0.01
NYHA II	632 (14.7)	120 (11.2)	158 (14.7)	191 (17.8)	163 (15.1)	
NYHA III	1,941 (45.1)	489 (45.5)	486 (45.1)	465 (43.2)	501 (46.6)	
NYHA IV	1,722 (40.0)	466 (43.3)	431 (40.0)	417 (38.8)	408 (37.9)	
Unknown	10 (0.2)	0 (0)	3 (0.3)	3 (0.3)	4 (0.4)	
HR, bpm/min (IQR)	85.0 (72.0–100.0)	85.0 (74.0–100.0)	84.0 (72.0–99.0)	84.0 (72.0–98.0)	86.0 (74.0–100.0)	0.05
SBP, mmHg (IQR)	130.0 (116.0–148.0)	127.0 (110.0–143.0)	130.0 (115.0–145.0)	130.0 (118.0–149.0)	134.0 (120.0–150.0)	<0.01
Albumin, g/L (IQR)	38.9 (35.9–41.9)	38.3 (35.2–41.2)	39.0 (35.7–41.8)	39.1 (36.3–42.3)	39.3 (36.5–42.5)	<0.01
Na, mmol/L (IQR)	140.0 (137.1–142.0)	139.3 (136.5–142.0)	140.0 (137.0–142.0)	140.0 (137.4–142.3)	140.9 (138.0–143.0)	<0.01
NT-proBNP, pg/mL (IQR)	1,423.0 (581.7–3,132.0)	2,126.5 (866.2–4,360.5)	1,667.0 (716.7–3,823.0)	1,237.0 (500.0–2,640.0)	970.1 (401.5–2,048.0)	<0.01
hs-cTnT, ng/L (IQR)	20.7 (12.4–38.2)	24.6 (14.7–46.8)	20.9 (12.7–39.2)	19.7 (11.7–36.2)	18.7 (11.1–34.0)	<0.01
eGFR, ml/min/1.73 m^2^ (IQR)	68.2 (53.3–83.8)	66.9 (50.8–83.1)	66.5 (52.0–81.2)	69.6 (56.5–84.4)	68.9 (54.3–86.1)	<0.01
LDL, mmol/L (SD)	2.4 ± 0.9	2.3 ± 0.9	2.3 ± 0.9	2.4 ± 0.8	2.4 ± 0.9	<0.01
TG, mmol/L (SD)	1.3 ± 1.0	1.1 ± 0.8	1.2 ± 0.7	1.4 ± 1.1	1.5 ± 1.1	<0.01
HDL, mmol/L (SD)	1.1 ± 0.3	1.2 ± 0.4	1.1 ± 0.4	1.1 ± 0.3	1.0 ± 0.3	<0.01
Waist circumference, cm (IQR)	89 (80–98)	84 (76–91)	87 (80–94)	90 (83–97)	96 (88–105)	<0.01
BMI, kg/m^2^ (IQR)	24.0 (21.4–26.7)	19.6 (18.4–21.0)	22.6 (21.6–23.9)	25.1 (24.2–26.1)	29.0 (27.3–31.2)	<0.01
FM index, kg/m^2^ (IQR)	7.5 (5.8–9.5)	5.8 (4.5–6.9)	7.3 (5.5–8.5)	8.2 (6.5–10.0)	10.2 (7.9–12.5)	<0.01
Smoking history, *n* (%)	1,102 (25.6)	265 (24.7)	282 (26.2)	286 (26.6)	269 (25.0)	0.7
**Comorbidities**, ***n*** **(%)**						
Metabolic syndrome	1,293 (30.0)	101 (9.4)	173 (16.0)	387 (36.0)	632 (58.7)	<0.01
Hypertension	2,512 (58.4)	520 (48.4)	607 (56.3)	641 (59.6)	744 (69.1)	<0.01
Cardiomyopathy	1,533 (35.6)	370 (34.4)	370 (34.3)	408 (37.9)	385 (35.8)	0.27
Atrial fibrillation	1,566 (36.4)	401 (37.3)	407 (37.8)	399 (37.1)	359 (33.4)	0.13
Valvular heart disease	695 (16.1)	213 (19.8)	197 (18.3)	161 (15.0)	124 (11.5)	<0.01
COPD	842 (19.6)	272 (25.3)	226 (21.0)	175 (16.3)	169 (15.7)	<0.01
Diabetes mellitus	1,360 (31.6)	268 (24.9)	319 (29.6)	382 (35.5)	391 (36.3)	<0.01
Anemia	960 (22.3)	321 (29.9)	275 (25.5)	201 (18.7)	163 (15.1)	<0.01
Coronary heart disease	2,476 (57.5)	619 (57.6)	640 (59.4)	631 (58.6)	586 (54.5)	0.1
Prior revascularization	707 (16.4)	159 (14.8)	188 (17.4)	190 (17.7)	170 (15.8)	0.22
**Medications at discharge**, ***n*** **(%)**						
ACEI/ARB	2,290 (53.2)	540 (50.2)	537 (49.8)	600 (55.8)	613 (57.0)	<0.01
β-blocker	2,602 (60.4)	630 (58.6)	640 (59.4)	678 (63.0)	654 (60.8)	0.17
MRA	2,766 (64.3)	730 (67.9)	677 (62.8)	674 (62.6)	685 (63.7)	0.03

*LVEF, left ventricle ejection fraction; NYHA, New York Heart Association; HR, heart rate; SBP, systolic blood pressure; hs-cTnT, high sensitivity cardiac troponin T; NT-pro BNP, N-terminal brain natriuretic peptide precursor; eGFR, estimated glomerular filtration rate; BMI, body mass index; FM index, fat mass index; COPD, chronic obstructive pulmonary disease; ACEI, angiotensin-converting enzyme inhibitor; ARB, angiotensin receptor blocker; MRA, mineralocorticoid receptor antagonist*.

### Prognosis Analysis

During the 1-year follow-up, 691 patients (16.1%) died. In the multivariate model, higher BMI was found to be associated with a lower risk of death. With the normal BMI group (BMI 18.5- 24 kg/m^2^) as a reference, HRs for BMI of <18.5, 24–28, and ≥28 kg/m^2^ were 1.39 [95% confidence interval (CI) 1.10–1.76], 0.74 (95% CI 0.62–0.89), and 0.71 (95% CI 0.55–0.94), respectively (*p* < 0.01 for trend).

**Figure 1:**
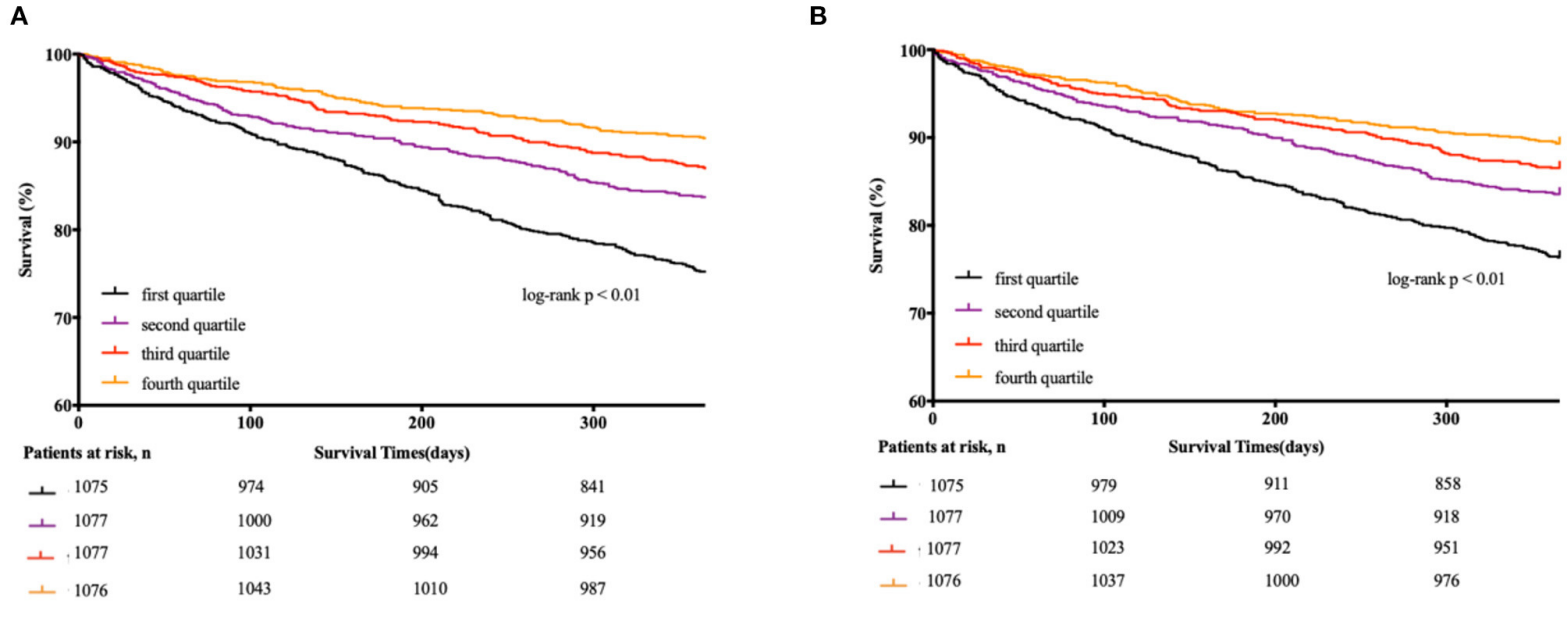
Kaplan-Meier estimates of 1-year all-cause mortality according to quartiles of each body component. **(A)** Kaplan-Meier curve according to quartiles of LBM index for the all-cause mortality. **(B)** Kaplan-Meier curve according to quartiles of FM index for the all-cause mortality.

The cumulative incidence of death by each LBM and FM index quartile is described using the Kaplan-Meier method in Figure 1. A significantly higher 1-year mortality was observed among patients with LBM index in the 1st quartile (24.6%), compared with 17.5, 12.1, and 9.8% for those in the 2nd, 3rd, and 4th quartile, respectively. And the 1-year mortality rates among patients with FM index in the 1st, 2nd, 3rd, and 4th quartile were 23.2, 16.6, 13.2, and 10.8%, respectively (both log-rank *p* < 0.01).

Table 2 shows the association between the LBM index quartiles and mortality. The higher quartile of LBM index was associated with lower mortality in all models. In the fully adjusted model (model 3), LBM index was still negatively correlated with death risk. Compared with participants in the 1st LBM index quartile, the risk of death was lower for those in the 2nd quartile (HR 0.80, 95% CI 0.66–0.97), the 3rd quartile (HR 0.65, 95% CI 0.52–0.83), and the 4th quartile (HR 0.61, 95% CI 0.45–0.82).

**Table 2:** Hazard ratios (HR) for 1-year mortality of HF patients by lean body mass (LBM) index quartiles.

**LBM index quartile**	Model 1^*^		**Model 2^†^**		**Model 3^‡^**	
	**HR (95%CI)**	***P*-value**	**HR (95%CI)**	***P*-value**	**HR (95%CI)**	***P*-value**
1	Ref.		Ref.		Ref.	
2	0.70 (0.58–0.84)	<0.01	0.79 (0.65–0.95)	0.01	0.80 (0.66–0.97)	0.03
3	0.48 (0.39–0.59)	<0.01	0.63 (0.51–0.79)	<0.01	0.65 (0.52–0.83)	<0.01
4	0.38 (0.30–0.47)	<0.01	0.57 (0.45–0.73)	<0.01	0.61 (0.45–0.82)	<0.01
*p*-value for trend	<0.01		<0.01		<0.01	

* *Model 1: Unadjusted*.

† *Model 2: Adjusted for age, sex, education level, systolic blood pressure at admission, heart rate at admission, NYHA class, LVEF, serum sodium, serum albumin, Hs-cTnT, NT-proBNP, eGFR, current smoking status, the history of coronary heart disease, hypertension, chronic obstructive pulmonary disease, anemia, valvular heart disease, diabetes mellitus, atrial fibrillation, the prescription of ACEI/ARB, β-blocker, and MRA*.

‡ *Model 3: Adjusted using characteristics for Model 2 by adding FM index*.

Table 3 shows the association between the FM index quartiles and mortality. Higher FM index quartile was associated with better survival without adjusting for LBM index (models 1 and 2), while the protective effect of FM was no longer significant after adjusting for LBM index (model 3).

**Table 3:** Hazard ratios (HR) for 1-year mortality of HF patients by fat mass (FM) index quartiles.

**FM index quartile**	Model 1^*^		**Model 2^†^**		**Model 3^‡^**	
	**HR (95%CI)**	***P*-value**	**HR (95%CI)**	***P*-value**	**HR (95%CI)**	***P*-value**
1	Ref.		Ref.		Ref.	
2	0.70 (0.58–0.85)	<0.01	0.81 (0.67–0.99)	0.04	0.88 (0.72–1.08)	0.23
3	0.56 (0.45–0.69)	<0.01	0.74 (0.60–0.91)	<0.01	0.86 (0.69–1.08)	0.20
4	0.47 (0.38–0.59)	<0.01	0.69 (0.54–0.87)	<0.01	0.92 (0.70–1.21)	0.55
*p*-value for trend	<0.01		<0.01		0.44	

* *Model 1: Unadjusted*.

† *Model 2: Adjusted for age, sex, education level, systolic blood pressure at admission, heart rate at admission, NYHA class, LVEF, serum sodium, serum albumin, Hs-cTnT, NT-proBNP, eGFR, current smoking status, the history of coronary heart disease, hypertension, chronic obstructive pulmonary disease, anemia, valvular heart disease, diabetes mellitus, atrial fibrillation, the prescription of ACEI/ARB, β-blocker, and MRA*.

‡ *Model 3: Adjusted using characteristics for Model 2 by adding LBM index*.

When accounting for LBM index and FM index as continuous covariates in the fully adjusted Cox model (model 3), each 1 SD increase in the LBM index decreased the risk of mortality (HR 0.77, 95% CI 0.67–0.84), while each 1 SD increase in the FM index did not decrease the risk of death (HR 0.95, 95% CI 0.83–1.09) (Table 4).

**Table 4:** Hazard ratios (HR) for 1-year mortality of HF patients according to a 1-SD increase in LBM index and FM index.

	Model 1^*^		**Model 2^†^**		**Model 3^‡^**	
	**HR (95%CI)**	***P*-value**	**HR (95%CI)**	***P*-value**	**HR (95%CI)**	***P*-value**
LBM index	0.72 (0.67–0.79)	<0.01	0.75 (0.67–0.84)	<0.01	0.77 (0.67–0.84)	<0.01
FM index	0.74 (0.67–0.81)	<0.01	0.82 (0.73–0.92)	<0.01	0.95 (0.83–1.09)	0.50

* *Model 1: Unadjusted*.

† *Model 2: Adjusted for age, sex, education level, systolic blood pressure at admission, heart rate at admission, NYHA class, LVEF level, serum sodium, serum albumin, Hs-cTnT, NT-proBNP, estimated glomerular filtration rate, current smoking status, the history of coronary heart disease, hypertension, chronic obstructive pulmonary disease, anemia, valvular heart disease, diabetes mellitus, atrial fibrillation, the prescription of ACEI/ARB, β-blocker, and MRA*.

‡ *Model 3: Adjusted using characteristics for Model 2 by adding FM index or LBM index*.

**Figure 2:**
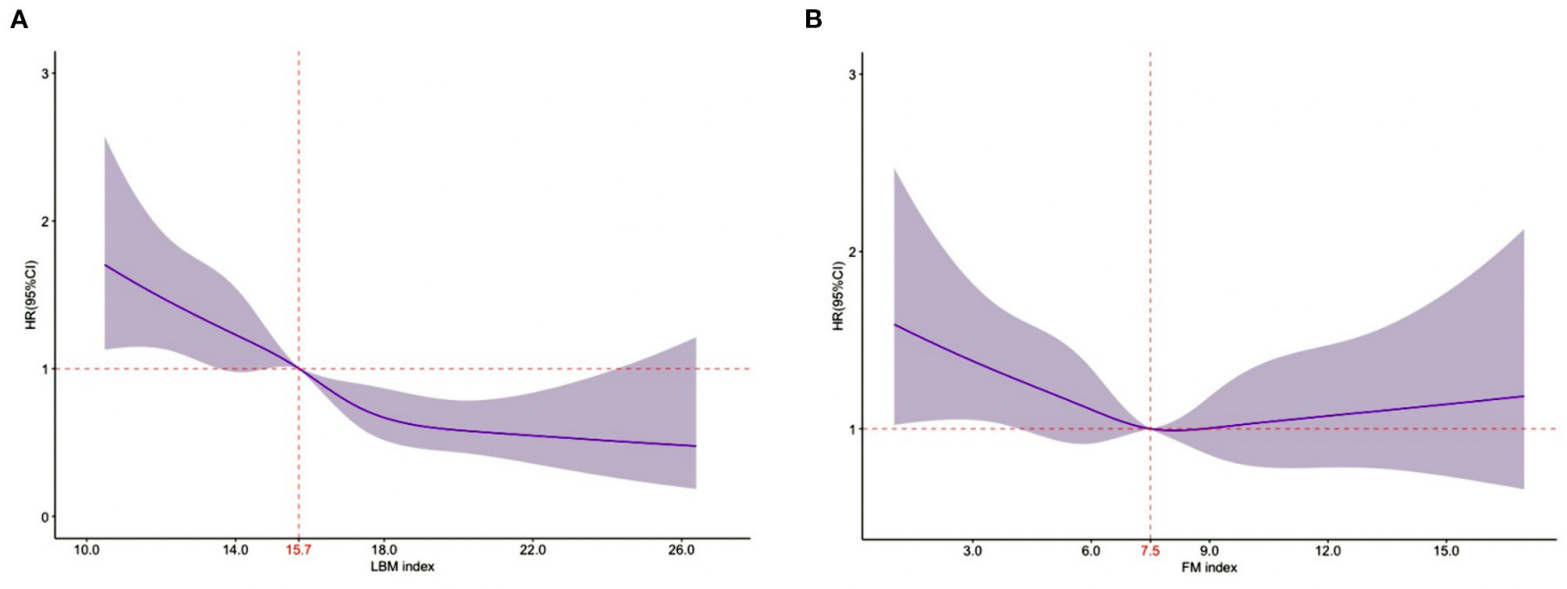
Relationship of the LBM index and the FM index with 1-year all-cause mortality using restricted cubic splines. **(A)**. Relationship between LBM index and 1-year all-cause mortality [the reference point was 15.7 (median); *P*-overall association < 0.01; *P*-non-linearity = 0.52]. **(B)**. Relationship between FM index and 1-year all-cause mortality [the reference point was 7.5 (median); *P*-overall association = 0.19; *P*-non-linearity = 0.22]. **(A,B)** represent the results of multivariate analyses adjusted for age, sex, education level, systolic blood pressure at admission, heart rate at admission, NYHA class, LVEF level, serum sodium, serum albumin, hs-cTnT, NT-proBNP, eGFR, current smoking status, the history of coronary heart disease, hypertension, chronic obstructive pulmonary disease, anemia, valvular heart disease, diabetes mellitus, atrial fibrillation, the prescription of ACEI/ARB, β-blocker, MRA, and mutually adjust for FM index or LBM index.

In the RCS analysis, LBM index was associated with mortality in a linear way, all-cause mortality decreased consistently with increasing LBM Index (*P*-overall association < 0.01; *P*-non-linearity = 0.52). However, no significant association between FM index and mortality was observed (*P*-overall association =0.19; *P*-non-linearity = 0.22) (Figure 2).

**Figure 3:**
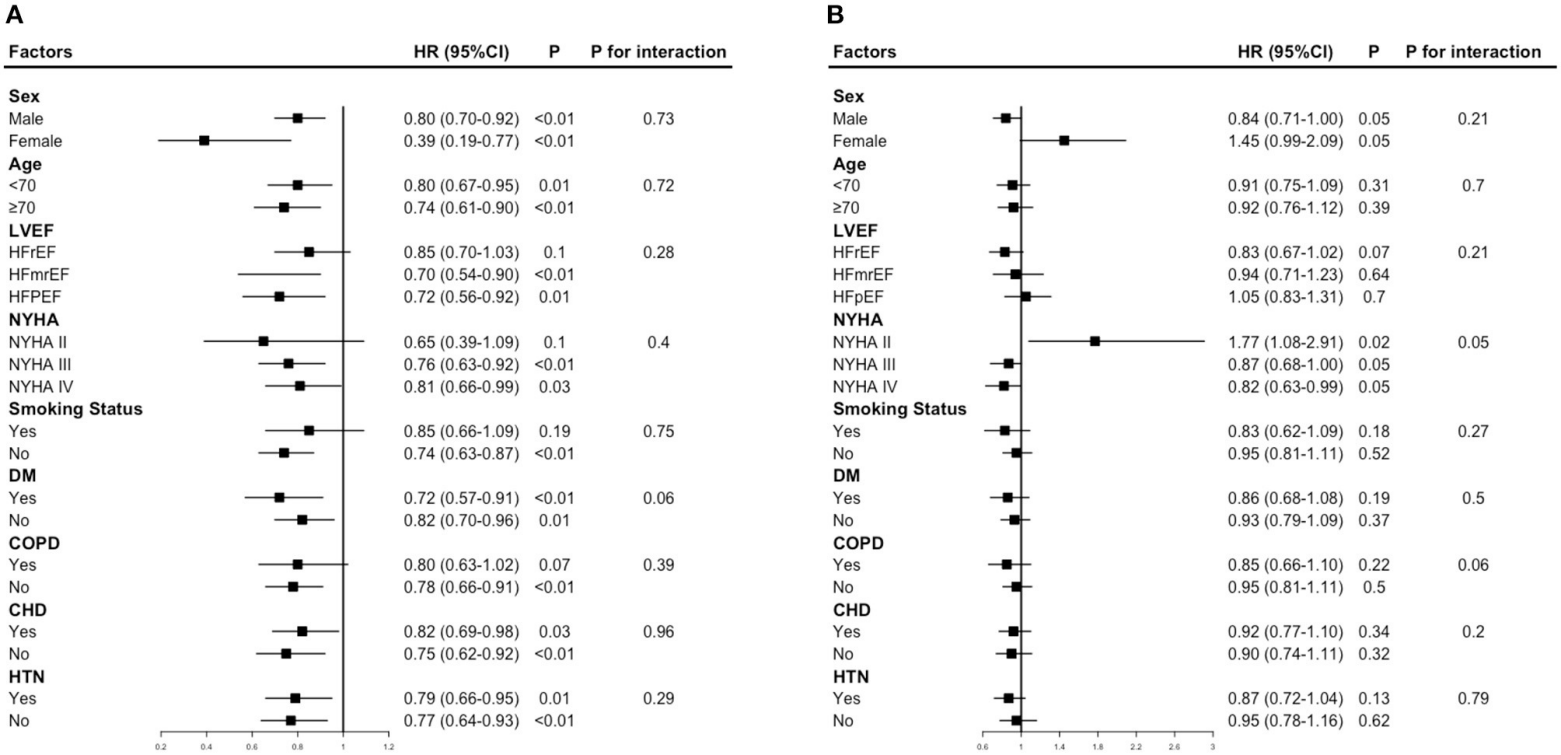
Hazard ratios for mortality according to a 1 -SD increase in LBM index or FM index. **(A)** Hazard ratios per 1 standard deviation–increase in LBM index for mortality. **(B)** Hazard ratios per 1 standard deviation–increase in FM index for mortality. Each stratification was adjusted for all factors in model 3, except for the stratification factor itself.

Figure 3 provides the results of the stratified analyses. The NYHA class modified the prognostic association of FM index and mortality (*P*-interaction = 0.05), higher FM index was strongly associated with poor prognosis among patients in NYHA II (HR 1.77, 95% CI 1.08–2.91), while associated with better survival among patients in NYHA III/IV (NYHA III: HR 0.87, 95% CI 0.68–1.00; NYHA IV: HR 0.82, 95% CI 0.63–0.99). No factor played an interactive role in the association between the LBM index and mortality.

Our results remained robust in several sensitivity analyses. The results did not change with the use of different categories for the LBM index and FM index (thirds or fifths) or with the use of prediction equations developed from Chinese population (Supplementary Tables 2–4). Analyses excluding patients who died during the first 3 months of follow-up or whose BMI was <18.5 kg/m^2^ yielded consistent findings compared with the results of the primary multivariate analyses (Supplementary Table 5).

## Discussion

In this large prospective cohort of patients hospitalized for HF in China, we demonstrated that LBM index was inversely related to mortality, while FM index was not associated with the risk of death.

We expand on previous literatures in several respects. Firstly, this is the first large-scale, nationwide study to assess the association between body composition and mortality among HF patients. To date, only a limited number of studies have examined this relation, and the findings were inconsistent (16–19). Although these studies used a direct measurement of body composition, the lack of statistical power that resulted from the small sample size (198–418 patients) makes it impossible to draw firm conclusions. Our study used previously validated anthropometric equations to estimate LBM and FM (14, 20), hence we could estimate the relation in a large-scale clinical epidemiological setting, and the further full adjustment for important covariates allows us to reveal robust conclusions.

Secondly, our study quantified the independent prognostic value of body constituents. Compared with previous studies, the mutual adjustment for LBM and FM in our study permits accurate analysis of the interplay of these variables' effects on survival. We found a survival benefit associated with high LBM that was independent of any effect of FM, while FM seems to be protective only if no adjustment was made for LBM. Prior studies evaluated the effect of fat on clinical outcomes without adjusting for muscle mass, and showed an protective effect of fat (16, 17, 25). Because higher FM in general is correlated with higher muscle mass (26), one could hypothesize that the lower mortality among patients with a high level of FM is a result of having large muscle mass. Accordingly, muscle mass could be a confounder when evaluating the association of FM and mortality, and it should be adjusted to tease out the independent effect of FM.

Thirdly, we present an in-depth analysis of the effect of body composition by stratified analyses, which has not been well-explored in previous studies. As shown in our study, the inverse association between LBM index and mortality was consistent in all subgroups. Increased muscle mass provides important metabolic benefits. Firstly, skeletal muscle is the primary target tissue for insulin-mediated glucose uptake, it has important role as an energy production and consumption system that influences the whole energy metabolism. Secondly, skeletal muscle could produce and secrete hundreds of myokines, like IL-15, BDNF and LIF, and these myokines were related with favorable changes in cardiometabolic profile, improved insulin sensitivity, anti-inflammation and antioxidant capacity (27, 28). Besides, muscle mass could indicate cardiorespiratory fitness to some extent (29), which is well-reported to be related with survival (30, 31). Hence, the result that muscle mass was universally favorable in all subgroups was not unexpected.

In addition, for the first time, we found that higher FM index exerts detrimental effect on mortality among HF patients in NYHA II, while it is associated with better survival in NYHA III/IV patients. The pathophysiologic mechanism of a relation between excess fat mass and increased mortality is well-established. Etiologic pathways include insulin resistance, inflammation and hormonal perturbations (32). Besides, obesity is an important risk factor for an expanding set of diseases, including diabetes mellitus, cardiovascular disease, which were all related with poor clinical outcomes (33). However, a growing number of studies support a survival benefit of adipose tissue in critical illness (34). And our study also indicated that higher FM index correlate with better survival in NYHA III/IV patients. The HF patients in NYHA III/IV are highly likely to suffer from cachexia, which is a sign of poor prognosis. It is reported that the symptoms (dyspnea, gut oedema), the neurohormonal and inflammatory activation in critical illness induce marked depletion of fat mass through enhanced fat catabolism, facilitating the development of cachexia (35). In this regard, among NYHA III/IV patients, the patients with high fat mass were less likely to develop cachexia, and these patients might have better prognosis than patients with low fat mass (36). The pathophysiologic mechanisms linking adipose to survival benefit in critical illness were proposed to be related with energy reserves, anti-inflammatory mediators, endotoxin-binding lipoproteins, cardioprotective metabolic effects (34). Apart from the above, our study revealed that patients with a higher level of FM tend to exhibit a higher mortality in women, while exhibit a lower mortality in men (both marginally statistically significant). One study used waist-to-hip ratio to assess obesity, and the result was largely consistent with our finding (37). The mechanism behind this association and difference based on gender has be speculated to be related with different hormones level and fat distribution (38). More research is warranted to validate our findings, and the exact mechanism need to be further evaluated in future studies. Besides, the gender and disease severity might be taken into consideration when designing clinical trials targeting obesity management in HF.

### Clinical Implications

Firstly, patients with the same BMI may have different body compositions and thus different risks of mortality. Therefore, measurement of body composition beyond total body mass should be incorporated in clinical assessments of HF patients to better identify patients at higher risk of death. Secondly, the predicted body composition indices estimated by anthropometric prediction equations, which are time-efficient and inexpensively measurable, can be integrated into routine clinical practice, especially in acute disease settings. Thirdly, in the era of precision medicine, the measurement of body composition could be used to formulate treatment recommendations. Patients with low muscle mass may benefit from more aggressive HF therapies, like β-blockers or ACEIs (39, 40), and additional treatment approaches to maintain or promote muscle mass through lifestyle modification (i.e., adequate protein supplementation, muscle-strengthening exercise) are important interventions to improve the clinical outcomes of HF patients (41–43). In addition, the recommendation of weight loss among obese HF patients should be considered more deliberately since the effect of fat mass might differ according to the disease severity.

### Limitations

Several limitations should be acknowledged in the interpretation of this study. Firstly, this was an observational study, and although we adjusted for important clinical covariates affecting prognosis, unmeasured or residual confounding factors may remain. Information on other prognostic factors, such as fitness ability and weight change, which may influence outcomes, was not available in this study. Secondly, using equations to calculate LBM and FM was not the most accurate way of measurements. However, the predictive ability for body compositions of these equations has been validated by other studies (14, 20). Besides, LBM and FM were analyzed in terms of categorical variables, making the exact value less important. Thirdly, reverse causality may in part be responsible for our observed associations. However, we minimized the potential for reverse causation due to advanced disease status by excluding patients who died within 3 months after discharge or with BMI < 18.5 kg/m^2^, and the conclusions remained unchanged.

### Future Directions

Firstly, further studies are needed to define the biologic mechanisms and relative importance of these mechanisms linking muscle mass to mortality, which would inform therapeutic approaches. Secondly, since the role of adipose tissue in HF prognosis is complex, more studies are warranted to evaluate the effect of different adipose tissue (visceral, subcutaneous, intermuscular, and intramuscular). Thirdly, few studies investigated the association between muscle-to-fat ratio and HF mortality, understanding how relative proportions of muscle mass and fat mass contribute to mortality will help identify which body composition phenotypes are optimal for survival in HF patients.

## Conclusion

Among patients hospitalized for HF, increased LBM, but not FM, predicts a lower risk of mortality.

## Data Availability Statement

The original contributions presented in the study are included in the article/Supplementary Materials, further inquiries can be directed to the corresponding authors.

## Ethics Statement

The studies involving human participants were reviewed and approved by the Ethics Committees of Fuwai Hospital and all collaborating hospitals. The patients/participants provided their written informed consent to participate in this study.

## Author Contributions

YG: concept, design, analysis, and drafting of the paper. JL, LZ, YG, BW, and XW: revision of the paper. XZ and JL: revision of the paper and responsible for the overall content as guarantor. All authors approved the submitted version of the manuscript.

## Funding

This work was supported by the China Academy of Chinese Medical Sciences Innovation Fund for Medical Science (2021-12M-1-009), the National Key Technology R&D Program (2015BAI12B02) from the Ministry of Science and Technology of China, and the 111 Project from the Ministry of Education of China (B16005).

## Conflict of Interest

The authors declare that the research was conducted in the absence of any commercial or financial relationships that could be construed as a potential conflict of interest.

## Publisher's Note

All claims expressed in this article are solely those of the authors and do not necessarily represent those of their affiliated organizations, or those of the publisher, the editors and the reviewers. Any product that may be evaluated in this article, or claim that may be made by its manufacturer, is not guaranteed or endorsed by the publisher.
