# Supplementary material for: Association of Lean Body Mass and Fat Mass With 1-Year Mortality Among Patients With Heart Failure
Source: Front Cardiovasc Med. 2022 Feb 28;9:824628. doi: 10.3389/fcvm.2022.824628 (PMC8918916; doi:10.3389/fcvm.2022.824628)
Supplement: Supplementary file 1 [file Data_Sheet_1.PDF]

Association of Predicted Lean Body Mass and Fat Mass with Mortality among Patients with Heart Failure

Supplementary Materials

Supplementary Tables 1. Anthropometric prediction equations for LBM and FM developed from the National Health and Nutrition Examination Survey

LBM	Men	19.363 + 0.001 * age (year) + 0.064 * height (cm) + 0.756 * weight (kg) − 0.366 * waist circumference (cm) − 1.007
	Women	−10.683 − 0.039 * age (year) + 0.186 * height (cm) + 0.383 * weight (kg) − 0.043 * waist circumference (cm) − 0.340
FM	Men	−18.592−0.009*age (year)−0.080*height (cm) + 0.226 * weight (kg) + 0.387 * waist circumference (cm) + 1.050
	Women	11.817+0.041 * age (year) − 0.199 * height (cm) + 0.610 * weight (kg) + 0.044 * waist circumference (cm) + 0.325

Supplementary Table 2. Hazard ratios (HR) for 1-year mortality of HF patients by FM index or LBM index tertiles

LBM index tertile	Model 1*		Model 2†		Model 3‡	
	HR (95%CI)	P value	HR (95%CI)	P value	HR (95%CI)	P value
1	Ref.		Ref.		Ref.	
2	0.63 (0.53-0.75)	<0.01	0.76 (0.64-0.91)	<0.01	0.78 (0.64-0.95)	0.01
3	0.39 (0.32-0.48)	<0.01	0.58 (0.47-0.72)	<0.01	0.57 (0.44-0.73)	<0.01
p value for trend	<0.01		<0.01		<0.01	
**FM index tertile**	**Model 1***		**Model 2†**		**Model 3‡**	
	HR (95%CI)	P value	HR (95%CI)	P value	HR (95%CI)	P value
1	Ref.		Ref.		Ref.	
2	0.67 (0.56-0.80)	<0.01	0.80 (0.67-0.96)	0.02	0.92 (0.75-1.12)	0.42
3	0.46 (0.46-0.67)	<0.01	0.78 (0.64-0.95)	0.02	1.05 (0.83-1.33)	0.71
p value for trend	<0.01		<0.01		0.99	

Model1*: Unadjusted.

Model2†: Adjusted for age, sex, education level, systolic blood pressure at admission, heart rate at admission, NYHA class, LVEF, serum sodium, serum albumin, Hs-cTnT, NT-proBNP, eGFR, current smoking status, the history of coronary heart disease, hypertension, chronic obstructive pulmonary disease, anemia, valvular heart disease, diabetes mellitus, atrial fibrillation, the prescription of ACEI/ARB, β-blocker, MRA.

Model3‡: Adjusted using characteristics for Model 2 by adding FM index or LBM index.

Supplementary Table 3. Hazard ratios (HR) for 1-year mortality of HF patients by FM index or LBM index quintile

LBM index quintile	Model 1*		Model 2†		Model 3‡	
	HR (95%CI)	P value	HR (95%CI)	P value	HR (95%CI)	P value
1	Ref.		Ref.		Ref.	
2	0.78 (0.64-0.96)	0.02	0.87 (0.71-1.07)	0.19	0.89 (0.72-1.09)	0.26
3	0.54 (0.43-0.67)	<0.01	0.67 (0.53-0.84)	<0.01	0.69 (0.54-0.88)	<0.01
4	0.41 (0.32-0.53)	<0.01	0.60 (0.46-0.77)	<0.01	0.63 (0.48-0.83)	<0.01
5	0.37 (0.29-0.48)	<0.01	0.59 (0.46-0.78)	<0.01	0.64 (0.46-0.89)	<0.01
p value for trend	<0.01		<0.01		<0.01	

FM index quintile	Model 1*		Model 2†		Model 3‡	
	HR (95%CI)	P value	HR (95%CI)	P value	HR (95%CI)	P value
1	Ref.		Ref.		Ref.	
2	0.81 (0.66-0.99)	0.05	0.93 (0.75-1.15)	0.49	0.99 (0.80-1.23)	0.96
3	0.61 (0.48-0.76)	<0.01	0.74 (0.59-0.94)	<0.01	0.85 (0.67-1.08)	0.18
4	0.52 (0.41-0.66)	<0.01	0.72 (0.56-0.92)	<0.01	0.87 (0.67-1.12)	0.28
5	0.45 (0.35-0.58)	<0.01	0.68 (0.52-0.89)	<0.01	0.94 (0.69-1.28)	0.71
p value for trend	<0.01		<0.01		0.43	

Model1*: Unadjusted.

Model2†: Adjusted for age, sex, education level, systolic blood pressure at admission, heart rate at admission, NYHA class, LVEF, serum sodium, serum albumin, Hs-cTnT, NT-proBNP, eGFR, current smoking status, the history of coronary heart disease, hypertension, chronic obstructive pulmonary disease, anemia, valvular heart disease, diabetes mellitus, atrial fibrillation, the prescription of ACEI/ARB, β-blocker, MRA.

Model3‡: Adjusted using characteristics for Model 2 by adding FM index or LBM index.

Supplementary Table 4. Hazard ratios (HR) for 1-year mortality of HF patients by FM index or LBM index quartiles (prediction equations of body composition developed from Chinese population)

LBM index quartile	Model 1*		Model 2†		Model 3‡	
	HR (95%CI)	P value	HR (95%CI)	P value	HR (95%CI)	P value
1	Ref.		Ref.		Ref.	
2	0.63 (0.52-0.76)	<0.01	0.74 (0.61-0.90)	<0.01	0.74 (0.98-0.93)	0.01
3	0.49 (0.40-0.60)	<0.01	0.65 (0.52-0.80)	<0.01	0.60 (0.45-0.80)	<0.01
4	0.36 (0.28-0.45)	<0.01	0.56 (0.43-0.71)	<0.01	0.45 (0.31-0.66)	<0.01
p value for trend	<0.01		<0.01		<0.01	

FM index quartile	Model 1*		Model 2†		Model 3‡	
	HR (95%CI)	P value	HR (95%CI)	P value	HR (95%CI)	P value
1	Ref.		Ref.		Ref.	
2	0.68 (0.56-0.82)	<0.01	0.79 (0.65-0.96)	0.02	0.94 (0.75-1.17)	0.55
3	0.56 (0.45-0.69)	<0.01	0.72 (0.58-0.90)	<0.01	1.04 (0.79-1.38)	0.77
4	0.45 (0.36-0.57)	<0.01	0.69 (0.54-0.88)	<0.01	1.29 (0.88-1.90)	0.19
p value for trend	<0.01		<0.01		0.68	

Model1*: Unadjusted.

Model2†: Adjusted for age, sex, education level, systolic blood pressure at admission, heart rate at admission, NYHA class, LVEF, serum sodium, serum albumin, Hs-cTnT, NT-proBNP, eGFR, current smoking status, the history of coronary heart disease, hypertension, chronic obstructive pulmonary disease, anemia, valvular heart disease, diabetes mellitus, atrial fibrillation, the prescription of ACEI/ARB, β-blocker, MRA.

Model3‡: Adjusted using characteristics for Model 2 by adding FM index or LBM index.

Supplementary Table 5. Association between body composition indices and mortality, excluding patients who died during the first 3 months of follow-up and patients with BMI<18kg/m2

LBM index quartile	Model 1*		Model 2†		Model 3‡	
	HR (95%CI)	P value	HR (95%CI)	P value	HR (95%CI)	P value
1	Ref.		Ref.		Ref.	
2	0.69 (0.53-0.89)	<0.01	0.75 (0.57-0.98)	0.03	0.76 (0.58-1.00)	0.05
3	0.55 (0.42-0.73)	<0.01	0.70 (0.52-0.93)	0.01	0.72 (0.53-0.98)	0.03
4	0.42 (0.32-0.57)	<0.01	0.60 (0.44-0.82)	<0.01	0.64 (0.44-0.92)	0.02
p value for trend	<0.01		<0.01		0.02	

FM index quartile	Model 1*		Model 2†		Model 3‡	
	HR (95%CI)	P value	HR (95%CI)	P value	HR (95%CI)	P value
1	Ref.		Ref.		Ref.	
2	0.79 (0.60-1.03)	0.08	0.89 (0.68-1.17)	0.40	0.96 (0.73-1.27)	0.77
3	0.63 (0.48-0.84)	<0.01	0.82 (0.61-1.09)	0.17	0.94 (0.68-1.28)	0.67
4	0.53 (0.39-0.71)	<0.01	0.72 (0.53-0.99)	0.04	0.89 (0.62-1.28)	0.53
p value for trend	<0.01		0.03		0.60	

Model1*: Unadjusted.

Model2†: Adjusted for age, sex, education level, systolic blood pressure at admission, heart rate at admission, NYHA class, LVEF, serum sodium, serum albumin, Hs-cTnT, NT-proBNP, eGFR, current smoking status, the history of coronary heart disease, hypertension, chronic obstructive pulmonary disease, anemia, valvular heart disease, diabetes mellitus, atrial fibrillation, the prescription of ACEI/ARB, β-blocker, MRA.

Model3‡: Adjusted using characteristics for Model 2 by adding FM index or LBM index.
